# Comparative Investigation of Nano-Sized Silica and Micrometer-Sized Calcium Carbonate on Structure and Properties of Natural Rubber Composites

**DOI:** 10.3390/polym16081051

**Published:** 2024-04-11

**Authors:** Nabil Hayeemasae, Siriwat Soontaranon, Abdulhakim Masa

**Affiliations:** 1 Department of Rubber Technology and Polymer Science, Faculty of Science and Technology, Prince of Songkla University, Pattani Campus, Pattani 94000, Thailand; nabil.h@psu.ac.th; 2 Research Unit of Advanced Elastomeric Materials and Innovations for BCG Economy (AEMI), Faculty of Science and Technology, Prince of Songkla University, Pattani Campus, Pattani 94000, Thailand; 3 Synchrotron Light Research Institute, Muang District, Nakhon Ratchasima 30000, Thailand; siriwat@slri.or.th; 4 Rubber Engineering and Technology Program, International College, Prince of Songkla University, Hat Yai, Songkhla 90110, Thailand

**Keywords:** natural rubber, composites, silica, calcium carbonate, mechanical properties

## Abstract

Fillers have been widely used in natural rubber (NR) products. They are introduced to serve as a strategy for modifying the final properties of NR vulcanizates. Silica and calcium carbonate (CaCO_3_) are among the fillers of choice when the color of the products is concerned. In this case, a special focus was to compare the vulcanizing efficiency of NR filled with two different filler types, namely nano-sized silica and micrometer-sized CaCO_3_. This study focused on the effects of the loading level (10–50 parts per hundred parts of rubber, phr) on the final properties and structural changes of NR composites. The results indicated that increased filler loading led to higher curing torques and stiffness of the rubber composites irrespective of the type of filler used. The better filler dispersion was achieved in composites filled with CaCO_3_ which is responsible for less polarity of CaCO_3_ compared to silica. Good filler distribution enhanced filler–matrix interactions, improving swelling resistance and total crosslink density, and delaying stress relaxation. The modulus and tensile strength of both composites also improved over the content of fillers. The CaCO_3_-filled composites reached their maximum tensile strength at 40 phr, exceeding, by roughly 88%, the strength of an unfilled sample. Conversely, the maximum tensile strength of silica-filled NR was at 20 phr and was only slightly higher than that of its unfilled counterpart. This discrepancy was ascribed to the stronger rubber–filler interactions in cases with CaCO_3_ filler. Effective rubber–filler interactions improved strain-induced crystallization, increasing crystallinity during stretching and reducing the strain at which crystallization begins. In contrast, large silica aggregates with poor dispersion reduced the overall crosslink density, and degraded the thermomechanical properties, tensile properties, and strain-induced crystallization ability of the NR. The results clearly indicate that CaCO_3_ should be favored over silica as a filler in the production of some rubber products where high performance was not the main characteristic.

## 1. Introduction

Natural rubber (NR) is one of the most widely utilized materials, used in a variety of industrial and household products. Its unique properties include high elasticity combined with high strength, and the ability of NR to crystallize when under extensional deformation provides it with self-reinforcement character [1]. For practical use in engineering applications, NR is cross-linked and reinforced with certain fillers. Among the different fillers, carbon black and silica are the most commonly used ones in the rubber industry [2]. Carbon black requires a lot of energy consumption in its production [3,4], and it is incompatible with products requiring bright colors; a white filler would be preferable in many products. Silica is one of the most commonly utilized non-black fillers in the rubber industry. It was discovered that the inclusion of silica gives rubber products a unique combination of adhesion, abrasion, and aging resistances, and a high tear strength [5]. Moreover, silica provides rubber composites with good abrasion resistance, low rolling resistance, and good wet traction, which are necessary characteristics of car tires [6,7]. To achieve the greatest reinforcement when employing silica as the filler, a silane coupling agent is generally incorporated in the compounds in order to improve filler dispersion and silica–rubber (or filler–matrix) interactions [8,9,10]. Unfortunately, the high cost of the silane coupling agent prevents it from being widely used. In addition, compounding silica with rubber requires a long processing time, high mixing temperature, consumes a lot of energy, and causes pollution to the environment [11]. An alternative filler that requires less energy consumption and is more environmentally friendly would be highly desired.

Calcium carbonate (CaCO_3_) is also one of the most important fillers in NR vulcanizates. It is an inexpensive, abundant, and reusable natural resource [12]. Moreover, this filler can also improve the physical properties of the composites, such as modulus, hardness, dimensional stability, and thermal resistance [13]. Lots of studies have been conducted to investigate the use of CaCO_3_ as a filler in the NR matrix. For instance, Arayapranee et al. [13] investigated the effects of three alternative fillers, namely rice husk ash (RHA), silica, and CaCO_3_, on the final properties of 75:25 NR/ethylene propylene diene rubber (EPDM) blends as the polymer matrix. It was found that loading and type of filler influenced the processability of the blends. The greatest tensile and tear strengths, as well as abrasion resistance, were obtained with silica. However, better processability and resilience properties were gained from using RHA and CaCO_3_ fillers in the blends. Poompradub et al. [14] investigated the reinforcing effects of CaCO_3_ obtained from cuttlebone, with a comparison to a commercial CaCO_3_ filler. They found that the mechanical properties of NR composites filled with CaCO_3_ from cuttlebone were comparable to those achieved using the commercial CaCO_3_. Moonchai et al. [15] investigated cure characteristics, mechanical properties, and morphology of NR composites filled with 50 phr of CaCO_3_. CaCO_3_ had decreased the curing time and increased the torque difference. The mechanical properties were found to be degraded due to poor dispersion of the CaCO_3_ in the NR matrix. Surya et al. [16] revealed a possibility to enhance the rubber–filler interactions of CaCO_3_ filler in NR by adding epoxidized natural rubber as a compatibilizer. Despite the various studies conducted, the effects of CaCO_3_ on the final properties of NR composites remain entirely unexplored.

To gain a further understanding of the effects of CaCO_3_ filler in NR composites, this study was conducted. The main objective of the present study is to comparatively investigate the processability, morphological, mechanical, dynamic mechanical, and stress relaxation properties of NR composites filled with either silica or CaCO_3_. Interactions between the rubber matrix and dispersed filler were assessed from equilibrium swelling tests. Furthermore, the influences of these two fillers on stretch-induced crystallization were also studied, in order to better understand their reinforcement aspects.

## 2. Materials and Methods

### 2.1. Materials

The STR 20 grade of NR was manufactured by Sri Trang Agro-Industry Plc. (Songkhla, Thailand). Zinc oxide (ZnO) and stearic acid were purchased from Global Chemical Co., Ltd. (Samut Prakan, Thailand), and Imperial Chemical Co., Ltd. (Bangkok, Thailand), respectively. 1,2-dihydro-2,2,4-trimethyl-quinoline (TMQ) was manufactured by Flexsys N.V. (Brussels, Belgium). Sulfur was purchased from Siam Chemical Co., Ltd. (Samut Prakan, Thailand), and N-cyclohexyl-2-benzothiazole sulfonamide (CBS) was manufactured by Flexsys America L.P. Charleston (Creve Coeur, MO, USA). CaCO_3_ with trade name Omyacarb-1T was supplied by Surint Omya Chemicals (Thailand) Co., Ltd. (Surint, Thailand). It has an average particle size of about 1 µm. Silica with trade name Ultrasil VN3 was manufactured by Evonik Industries AG (Essen, Germany). It had a primary particle size of about 14 nm. All these materials and chemicals were used as received.

### 2.2. Sample Preparation

NR composites filled with various quantities of either silica or CaCO_3_ were prepared by using an internal mixer (HAAKE PolyLab OS, Thermo Scientific, Waltham, MA, USA) with 379 cm^3^ of mixing chamber volume. The Banbury-type rotor was used in this study. The NR and other ingredients shown in Table 1 were compounded at a rotor speed of 60 rpm and an initial temperature of 40 °C. The NR was first masticated for 2 min before adding stearic acid, ZnO, and TMQ. After 3 min, filler was added in alternating fashion and mixed for 3 min. The CBS was added and mixed for 1 min. Finally, the S was added. Total mixing time was fixed at 10 min for all the prepared blends. The dump temperature was about 135–140 °C. Prior to vulcanization, the rubber compounds were left at ambient room temperature for 24 h. The compounds were compression molded at 170 °C, each for its respective curing time, to obtain 2 mm thick crosslinked sheet samples. The rubber composites containing silica or CaCO_3_ fillers were noted as silica X or CaCO_3_ X, where X referred to the content of filler. The rubber without incorporation of filler was assigned as control.

### 2.3. Characterizations

#### 2.3.1. Rheometric Testing

NR composite compounds containing one of the two alternative fillers were evaluated for their curing properties using a moving die rheometer, Montech MDR 3000 BASIC (Buchen, Germany). The tests were run at 170 °C with a fixed oscillation angle of +/−0.5° and a frequency of 1.67 Hz for 30 min. The characteristics related to curing, including minimum torque (M_L_), maximum torque (M_H_), torque differential (M_H_ − M_L_), and cure rate index (CRI) are reported.

#### 2.3.2. Morphological Characteristics

Scanning electron microscopy (SEM), FEI Quanta 400FEG (Eindhoven, The Netherlands) was used to investigate the dispersion of filler in the composites. The sample was fractured in liquid nitrogen and gold-coated prior to the SEM imaging.

#### 2.3.3. Equilibrium Swelling Testing

An equilibrium swelling test was carried out to determine the swelling characteristics and estimate the crosslink densities of the rubber composites. Vulcanizates with and without fillers were immersed in toluene and kept in the dark for 3 days at room temperature. The swollen samples were then gently wiped to remove liquid from the surfaces and weighed. The swollen sample was heated at 70 °C to remove the solvent, continuing until constant weight. The degree of swelling was calculated as follows:

(1) $$\mathrm{Swelling}(\%)=\left(\frac{W_{s}-W_{i}}{W_{i}}\right)\times 100$$

where W_i_ is the initial (or dry) weight of sample (g) and W_s_ is the weight of swollen sample (g).

The crosslink density (ν) was estimated based on the Flory–Rehner equation [17]:

(2) $$\nu =-\frac{\ln(1-V_{r})+V_{r}+\chi {V_{r}}^{2}}{2\rho V_{s}\left({V_{r}}^{1/3}-\;0.5V_{r}\right)}$$

where χ is the polymer–solvent interaction parameter (here equal to 0.39 for the NR-toluene system [18]), ρ is density of the rubber (0.93 g/cm^3^), vs. is molar volume of the solvent, and V_r_ is the volume fraction of rubber in the swollen mass that can be calculated from Equation (3).

(3) $$V_{r}=\frac{\frac{W_{d}}{\rho_{r}}}{\begin{array}{c} \frac{\begin{array}{c} W_{d} \end{array}}{\rho_{r}}+\frac{W_{s}-W_{d}}{\rho_{s}} \end{array}}$$

where W_d_ is the de-swollen weight, W_s_ is the swollen weight, ρ_r_ is the density of rubber, and ρ_s_ is the density of solvent.

#### 2.3.4. Rubber–Filler Interactions Testing

The rubber–filler interaction parameter (Q_f_/Q_g_) was determined from the swelling measurements. The Q_f_/Q_g_ was calculated by applying the Lorenz–Park equation [19]:

(4) $$\frac{Q_{f}}{Q_{g}}=ae^{-z}+b$$

where f and g indices stand for gum and filled vulcanizates, a and b are constants, and z represents the weight fraction of filler, whereas Q represents the volume of solvent absorbed defined by [20]:

(5) $$Q=\frac{Swollen\;weight-Dreid\;weight}{\begin{array}{c} Original\;weight\times 100/Formular\;weight \end{array}}$$

#### 2.3.5. Temperature Scanning Stress Relaxation (TSSR) Testing

The stress relaxation behaviors of NR samples in both isothermal and non-isothermal conditions were recorded using a temperature scanning stress relaxation (TSSR) device (Brabender, Duisburg, Germany). The dumbbell-shaped specimen was cut in compliance with ISO 527 [21], type 5A, prior to clamping and pre-stretching at 50% strain and 23 °C. The samples were kept under this condition for 2 h and their isothermal stress relaxations were recorded. Afterward, the specimens were heated steadily at a rate of 2 °C/min until the test was completed and non-isothermal relaxations were recorded. Thermomechanical characteristics including the initial stress (σ_0_), and the temperatures at which the force has dropped by 10%, 50%, and 90% (T_10_, T_50_, and T_90_) from the initial force (F_0_) were recorded for each tested composite.

#### 2.3.6. Tensile Testing

A universal tensile testing apparatus, LR5K Plus (LLOYD Instruments, Bognor Regis, UK) was used to examine the tensile response characteristics of the various types of samples. The NR and its composite sheets were cut into dumbbell-shaped specimens using die type 2 (ISO 37 [22]). The test was run at room temperature with a crosshead speed of 500 mm/min.

#### 2.3.7. Dynamic Mechanical Property Testing

The dynamic mechanical property test was run in tension mode over the temperature range from −80 °C to 80 °C, with a heating rate of 2 °C/min, a frequency of 10 Hz, and a strain amplitude of 0.2% using a DMA 1 (Mettler Toledo, Greifensee, Switzerland). Storage modulus (E′), tan delta peak height (tan δ_max_) and glass transition temperature (T_g_) are reported.

#### 2.3.8. Wide-Angle X-ray Scattering (WAXS) Testing

Wide-angle X-ray scattering (WAXS) measurements were performed at the Siam Photon Laboratory, Synchrotron Light Research Institute, Nakhon Ratchasima, Thailand, to observe the changes in crystallinity during stretching for the various NR sample types. The test was performed at room temperature using 500 mm/min crosshead speed. All the WAXS data were normalized and validated by the SAXSIT software version: 4.60.

The changes in crystallinity (%) corresponding to the (200) and (120) planes during stretching were estimated using the following equation [23]:

(6) $$\mathrm{Crystallinity}\;(\%)=\frac{A_{cryst}}{A_{cryst}+A_{amor}}$$

where $A_{cryst}$ and $A_{amor}$ denote the area below the 200 and 120 crystalline peaks, and the amorphous halo area, respectively.

The average crystallite size corresponding to the (200) plane ($L_{200}$) was estimated from the Scherrer equation [1,24]:

(7) $$L_{200}=\frac{\kappa \lambda }{\beta_{1/2}cos\theta }$$

where λ is the wavelength, θ is Bragg angle, $\beta_{1/2}$ is the full width at half maximum of the diffraction peak, and κ is a constant.

## 3. Results

### 3.1. Curing Characteristics

**Figure 1:**
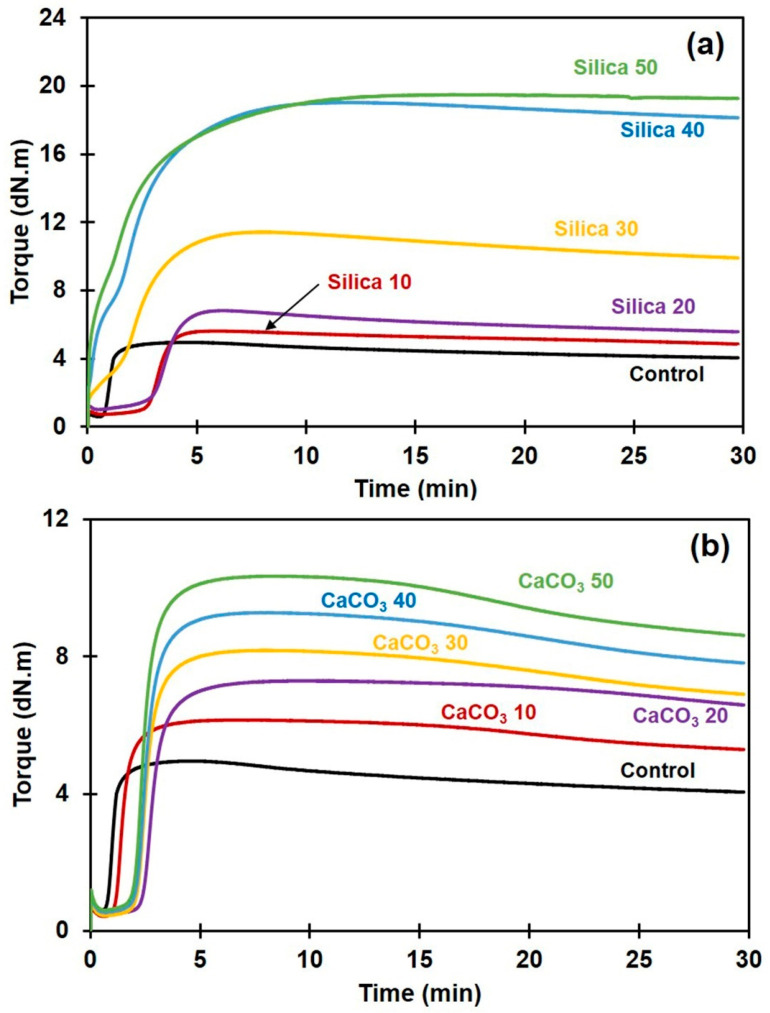
Curing characteristics of rubber composites filled with (**a**) silica or with (**b**) CaCO_3_.

Figure 1a,b show the curing characteristics of the NR and its composites filled with either silica or CaCO_3_. It can be seen that the development of torque in all NR composites was greatly affected by the type and content of the filler. The torque tended to increase with filler content irrespective of filler type. The filler caused a delay in the induction period (time before vulcanization starts), probably due to both types of filler absorbing the accelerator that initiates crosslinking reactions [5]. However, a different behavior of the induction period was noted for the silica-filled NR compounds when the filler loading was greater than 20 phr. This was tentatively attributed to the competition from silica–silica network formation [8]. An aggregation or clustering of silica particles within the rubber matrix has led to the formation of large, interconnected clusters or agglomerates which is called flocculation. Flocculation affected the pre-crosslinking of NR which can be viewed from the leverage of torque development at the induction period.

The cure characteristics in term of M_L_, M_H_, M_H_ − M_L_, and CRI are summarized in Table 2. The M_L_ refers to the initial viscosity of the compound. It is usually used as an indication of processability of the rubber compound [25]. The M_L_ of the composite compounds drastically increased with silica loading, while it decreased slightly with incorporation of CaCO_3_. This clearly suggests that the processability of the compounds filled with silica is more difficult and complicated than that of the CaCO_3_-filled ones. M_H_ is a measurement of stiffness or shear modulus of the fully vulcanized sample, and it increased significantly with filler content. This is due to the hardness of the filler and also due to it reducing the chain mobility of the rubber macromolecules [26]. The dispersed filler particles adsorbed some rubber chains on their surfaces, forming physical interactions, shortening the free segments of network chains, and decreasing the number of entanglements between consecutive crosslinks. Therefore, the stiffness of rubber increased with filler loading.

With either filler type, the torque difference M_H_ − M_L_ of the composites increased with filler loading, showing that the rigidity of rubber composites increased. There are two possible explanations for this effect. On one hand, the increase in M_H_ − M_L_ was due to an increased crosslink density as a result of rubber–filler interactions in addition to the chemical crosslinks. M_H_ − M_L_ is known to be indicative of the crosslink density in rubber composites [27]. On the other hand, the increase in M_H_ − M_L_ can partly be attributed to the filler–filler interactions, in particular when silica was used as the filler. Silica contains many silanol groups, and these react with each other by hydrogen bonding, forming filler–filler interactions. It has been reported that the silica filler networks among silica agglomerates formed by strong hydrogen bonds during vulcanization drastically increased vulcanization torque [8]. This may be associated with the poor filler dispersion and poor rubber–filler interactions in the silica-filled NR, as will be discussed in the next section. Other studies have also suggested that the increase in M_H_ − M_L_ is not only attributable to crosslink density but also to the filler–filler and the filler–rubber interactions [26].

The CRI of various composite samples decreased with filler content, suggesting that the crosslinking reactions were retarded by silica or by CaCO_3_. A decreased CRI in both cases was probably due to these fillers having a tendency to react with the curing accelerator, reducing the amount of accelerator that would promote the curing reactions, as reported earlier [5,28]. Compared to the composites filled with CaCO_3_, the decrease in CRI was more pronounced for silica-filled NR composites. This is due to the strongly acidic nature of silica, having a lot of chemically reactive silanol groups on the particle surfaces. These polar silanol groups adsorbed or otherwise reacted with the accelerator for the sulfur-based curing system, because the majority of accelerators are alkaline [5,17,29], reducing the quantity and efficiency of the accelerator in the vulcanization process and thereby decreasing the CRI.

### 3.2. Morphological Properties

**Figure 2:**
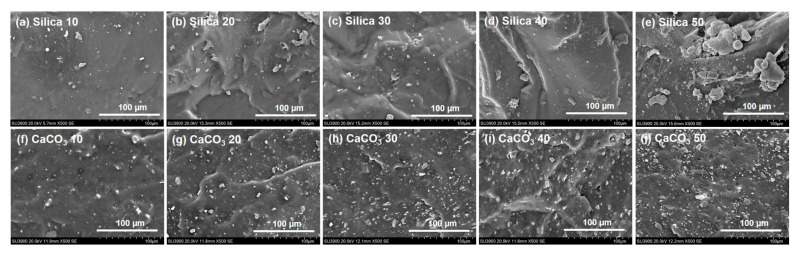
SEM micrographs of rubber composites filled with silica and CaCO_3_.

To gain more detailed information on the dispersion of the fillers by their loadings in the NR matrix, SEM imaging was performed, and the results are shown in Figure 2. It is generally seen that the morphology of rubber composites comprised filler particles dispersed in the rubber matrix. The size and dispersion level of the filler varied by both loading and type of filler. Compared to the CaCO_3_-filled NR samples, the morphology in silica-filled NR clearly exhibited larger agglomerates and their size increased with silica loading (Figure 2a–e). The silica agglomerate size was about 2.5–5 µm at 10 phr and about 5–30 µm at 50 phr. This was because of the large number of highly polar silanol groups on the silica surfaces. These silanol groups can form hydrogen bonds among themselves, forming strong filler–filler interactions [8,30]. Another observation was that the morphology of silica-filled rubber also revealed a poor filler distribution with large-sized agglomerates, confirming the significant filler–filler interactions. Agglomeration of silica has been generally observed in composites filled with silica, even with a silane coupling agent. It has been reported that only a small portion (~30%) of the silanol groups on the silica surfaces interact with the coupling agent, while the remaining (~70%) are unreacted [31]. This could be the reason why silica particles prefer to form large aggregates even at a low filler concentration. Better dispersion of filler with more uniformity and smaller size of aggregates (about 1–10 µm) was observed in the NR filled with CaCO_3_ (Figure 2f–j). The findings definitely demonstrate a difference in the abilities of these two fillers to agglomerate. One possible explanation for the lower propensity of CaCO_3_ to form aggregates compared to silica is that its particles lack active hydroxyl groups.

### 3.3. Equilibrium Swelling and Crosslink Density

**Figure 3:**
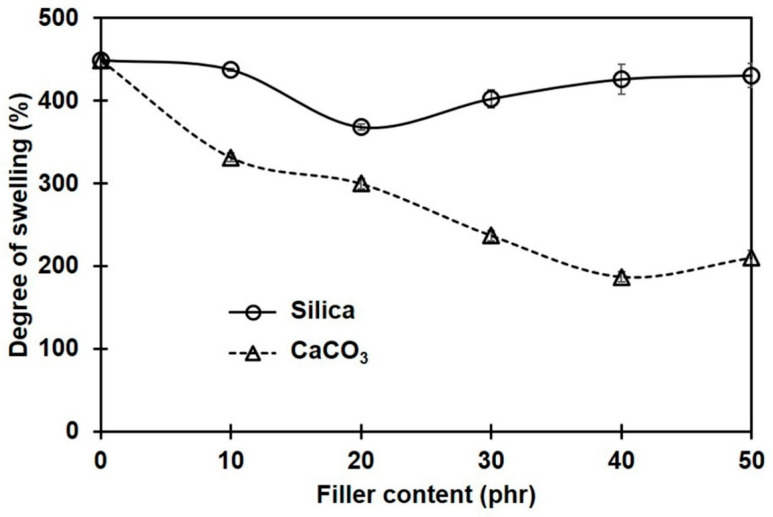
Swelling percentages of rubber composites filled with silica and CaCO_3_.

Figure 3 shows the percentage of swelling for NR and its composites containing different filler types and loadings. Regardless of the type of filler, the degree of swelling decreased with filler loading. This means that the filler limited the penetration of the toluene solvent into the NR composites. The lesser swelling of the rubber composites was due to the filler particles serving as obstacles to the diffusing solvent molecules [32] and to the rubber–filler interactions [33]. In comparison to the composites filled with silica, the swelling degrees of the composites containing CaCO_3_ were smaller. This was probably due to the better dispersion of CaCO_3_ filler, with smaller phase domain size and more uniformity in the NR matrix. The smaller size of the filler promotes better filler–rubber interactions in the composites [34,35]. As a result of the greater filler–rubber interactions, the molecular movements of rubber were hindered, making it more difficult for toluene to pass through the rubber matrix.

**Figure 4:**
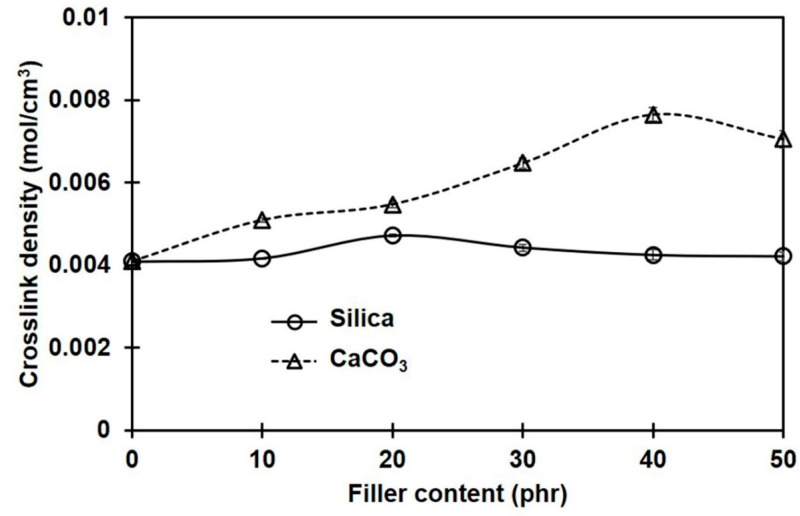
Crosslink densities in rubber composites filled with silica and CaCO_3_.

Considering the apparent crosslink densities of NR composites filled with different filler types and contents, these were determined from the Flory–Rehner equation and are shown in Figure 4. The crosslink density in the sample without filler is also included for comparison. Generally, the overall crosslink density increased with filler loading, and the choice of filler type significantly impacted the magnitude of the changes. Compared to silica-filled cases, the overall crosslink density in composites filled with CaCO_3_ was greater. The filler particles may act as additional crosslinking sites interacting physically and/or chemically with the rubber molecules, having strong bonding between the filler and the rubber matrix and thus increasing the crosslink density [4].

It should be noted that the crosslink density estimates from swelling equilibrium analysis and the curing property test (M_H_ − M_L_) were contradictory among the silica-filled composites. The crosslink density assessed from M_H_ − M_L_ had an increasing trend, whereas the swelling results showed the opposite trend. This difference can be explained by the different testing conditions. The curing test was conducted at a higher temperature (170 °C), and the torque generated would be related to the chemical crosslinks [36]. In the case of silica-filled rubber compounds, the formation of filler networks (filler–filler interactions) served as a competitive reaction, contributing to an increase in torque during vulcanization [8]. Thus, a drastic increase in torque was observed. In contrast, the crosslink density was estimated through the swelling coefficient at room temperature. Therefore, the effects of physical crosslinks, i.e., chain entanglements and filler–rubber interactions, were surely included in these results, in addition to the chemical crosslinks and filler–filler interactions. It is suggested that if there is an additional bonding between the filler and the rubber matrix, a stronger network would be formed at this stage. The extent of these linkages can be reflected in the crosslink density that can be determined from a swelling test [37]. Based on these results, stronger interactions between filler and rubber can be assumed in the CaCO_3_-filled composites compared to the silica-filled ones. The greatest crosslink density among the silica-filled composites was achieved at 20 phr, while the maximum was at 40 phr for CaCO_3_ filler. These might be the best loadings for the greatest mechanical property improvements, when silica or CaCO_3_ is used to fill NR composites.

### 3.4. Rubber–Filler Interactions

**Figure 5:**
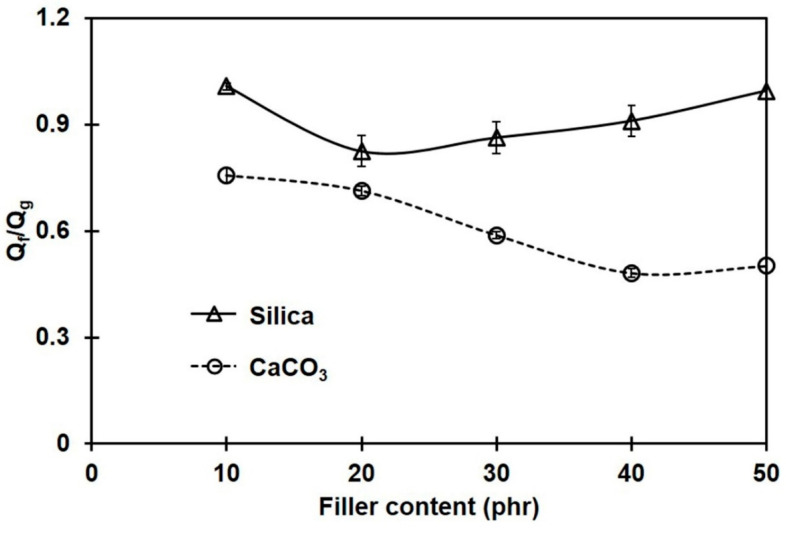
Interaction parameter (Q_f_/Q_g_) for the rubber composites filled with silica or CaCO_3_.

To confirm the extent of interactions between rubber and filler, Lorenz–Parks model was employed [19]. According to the Lorenz–Park relationship, the lower the interaction parameter, the higher the rubber–filler interactions [19,38]. Figure 5 shows Q_f_/Q_g_ obtained for the NR composites filled with different filler types and contents.

Obviously, Q_f_/Q_g_ continuously decreased with CaCO_3_ loading, while it slightly decreased in the silica-filled composites at a low filler loading, and then increased when the silica content was more than 20 phr; however, Q_f_/Q_g_ was still below that of the unfilled sample. This suggests that great silica–rubber interactions may be obtained only when the silica loading is less than 20 phr. The increase in agglomerate size of silica above 20 phr hinders the rubber–filler interactions. Since the Q_f_/Q_g_ values of the CaCO_3_-filled composites were lower than those of the silica-filled ones, the stronger rubber–filler interactions of CaCO_3_ with NR are confirmed. The rubber–filler interactions in the composites containing CaCO_3_ increased with the amount of filler, resulting in a reduction in NR chain mobility and limiting the penetration of a solvent throughout the rubber matrix. The greatest rubber–CaCO_3_ interactions were seen at 40 phr, which agreed well with the crosslink density. In contrast, the greater Q_f_/Q_g_ for the composites filled with silica implies fewer interactions between the silica filler and the rubber matrix. Similar behavior was also reported in [34].

### 3.5. Stress Relaxation

**Figure 6:**
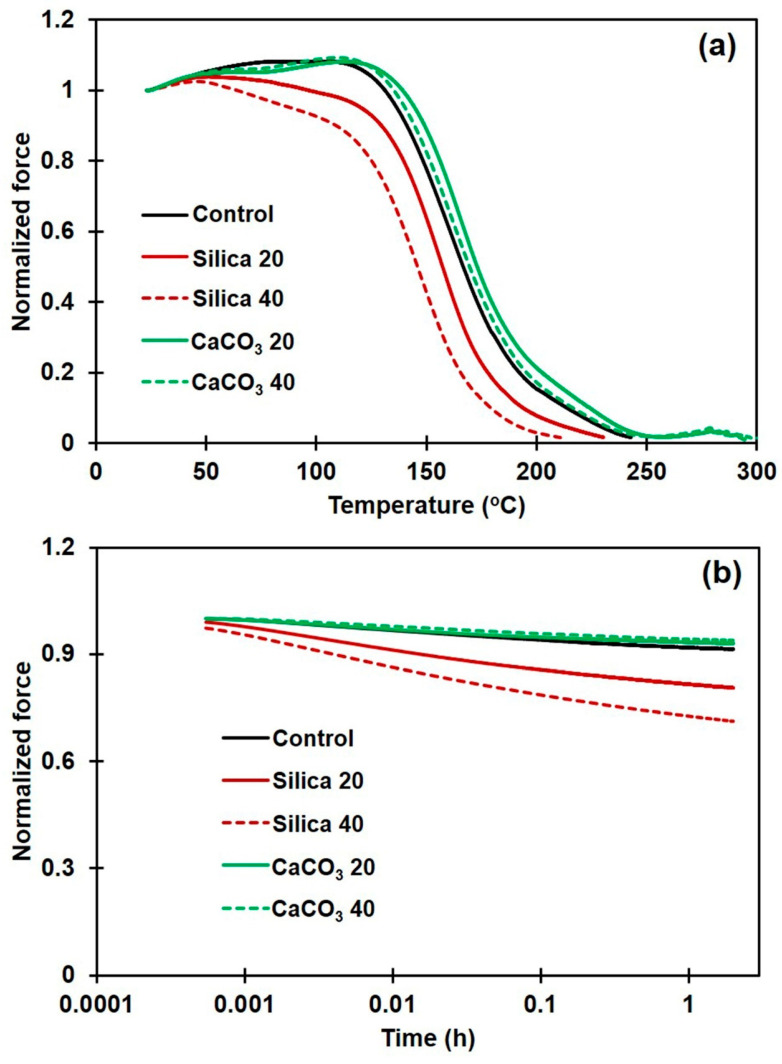
Normalized force during (**a**) non-isothermal, and (**b**) isothermal relaxation of rubber composites filled with silica or with CaCO_3_.

TSSR measurements during isothermal and non-isothermal conditions were performed to assess the thermomechanical properties and relaxation behaviors of the various composites. Figure 6a shows normalized force curves for NR and its composites with different filler types and loadings during non-isothermal conditions. In the temperature range 30–50 °C, the normalized force slightly increased due to an entropy effect [39,40]. A decrease in the normalized force over 50–100 °C was due to the breakdown of physical bonds between rubber molecules, or between rubber and filler [26,40]. Thus, a larger reduction in normalized force in this region would be attributed to the debonding of weak rubber–filler interactions, and this is seen in the silica composites. A steep decrease in normalized force above 100 °C was due to the cleavage of sulfur linkages between rubber chains (90–160 °C) and chain scission (160–220 °C) [41]. The raw data on thermomechanical properties obtained from the TSSR measurements, including σ_0_, T_10_, T_50,_ and T_90_ are summarized in Table 3 for all the NR composite samples.

From Figure 6a and Table 3, the incorporation of a filler greatly affected the stress relaxation behavior of the NR. Compared to the unfilled NR, the σ_0_ of composites containing 20 and 40 phr CaCO_3_ were clearly increased by about 29% and 52%, respectively, while the composites made with silica showed only small changes. These increases revealed the presence of additional linkages and networking between filler and rubber. Aside from the hydrodynamic effects, the rubber–filler interactions also partially contributed to the increase in σ_0_ of the rubber composites [26]. Furthermore, an increase in normalized force above 100 °C and the results on T_10_, T_50,_ and T_90_ for CaCO_3_-filled NR composites, compared to those for the unfilled NR and the silica composites, evidences strong interactions between the CaCO_3_ filler and the NR matrix. Conversely, the decrease in those values seen for the silica-filled NR demonstrates poor interactions between silica and NR. Figure 6b shows normalized forces for the NR composites containing different filler types during isothermal relaxation, and the slope values are shown in Table 3. This is another method for quantifying the interactions between rubber and filler. It is commonly acknowledged that the interactions between the rubber chains and the filler particles have an impact on the stress relaxation rate in a rubber composite. Stronger interactions are generally correlated with a lower relaxation rate [30,42]. A higher rate of stress decay was observed for silica-filled NR than for the CaCO_3_-filled one. For example, the negative slopes of the composites containing 20 and 40 phr CaCO_3_ were 0.0086 and 0.0092, respectively, while these were 0.0253 and 0.0371 for the composites with 20 and 40 phr silica, respectively. Compared to unfilled NR, a lower rate of stress relaxation was noticed in the composites filled with CaCO_3_ while the silica-filled NR composites showed greater relaxation slopes. A larger negative slope found for the silica-filled NR was probably due to the release of rubber chains that were poorly attached to the silica surfaces. On the other hand, stronger interactions between the rubber chains and the CaCO_3_ were generated due to the more uniform CaCO_3_ dispersion in the NR composites, resulting in delayed stress relaxation.

### 3.6. Tensile Properties

**Figure 7:**
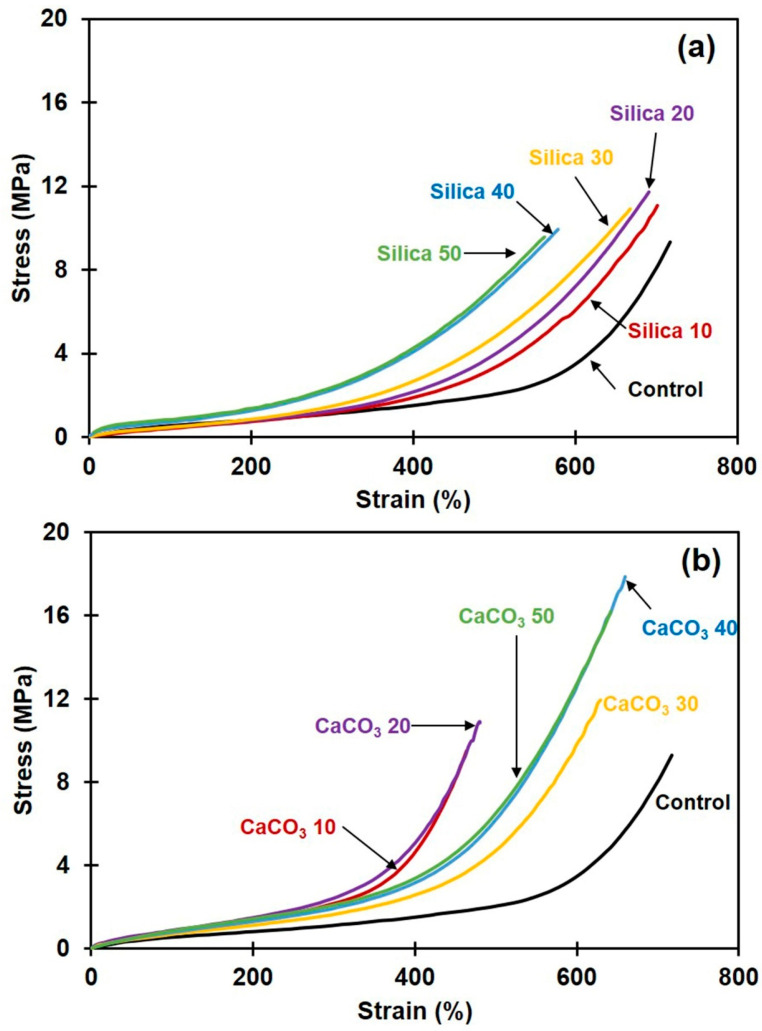
Stress-strain curves for rubber composites filled with (**a**) silica or with (**b**) CaCO_3_.

Figure 7a,b show representative stress-strain curves of the composites filled with silica and CaCO_3_, respectively. The stress-strain relation for unfilled NR is also included for comparison. Averages of 100% modulus (100M), tensile strength (TS), and elongation at break (EB) are summarized in Table 4 for all sample types. It is seen that the incorporation of filler increased 100M and TS of the NR composites, regardless of the type of filler. The increase in 100M was attributed to the filler particles being stiffer than rubber, which diminishes flexibility and increases the rigidity of the filled composites [43]. Compared to silica, the incorporation of CaCO_3_ provided greater increases in 100M and TS. This was attributed to the better filler dispersion and the better rubber–filler interactions, as previously suggested by morphological, swelling, and stress relaxation results. The improvement of tensile properties in case of CaCO_3_-filled NR was reported to be due to its good dispersion [44]. This phenomenon was evidently seen in SEM images (see Figure 2f–j). In addition to this, Jong [45] reported that that CaCO_3_ can interact with rubber chains through the non-rubber parts (protein containing in the NR) [45], improving the tensile properties. The greatest TS increase among the CaCO_3_-filled NR cases was achieved at 40 phr loading, with about 88% improvement over an unfilled sample. This is probably the optimum content of CaCO_3_ for obtaining the largest tensile property, also giving the highest crosslink density and strongest rubber–filler interactions (Figure 4 and Figure 5). Such a considerable improvement could not be observed without effective rubber–filler interactions. In the cases of NR filled with silica, the highest TS was about 20% over the unfilled NR, found at 20 phr. Further increase in the silica loading diminished the TS due to silica aggregation, as previously demonstrated in Figure 2. The aggregates reduce the filler–rubber interactions [46] and usually act as points of stress concentration [13], so the composites prematurely break and thus the TS decreases. Considering the EB of all composites, it was reduced by the incorporation of either silica or CaCO_3_ filler. The introduction of filler typically decreases rubber elasticity, which can be attributed to either inadequate filler dispersion, where agglomerates act as stress-concentrating imperfections, or to good rubber–filler interactions, which limit the movements of rubber chains [47]. Similar observations were also reported in [9,13].

### 3.7. Dynamic Mechanical Properties

**Figure 8:**
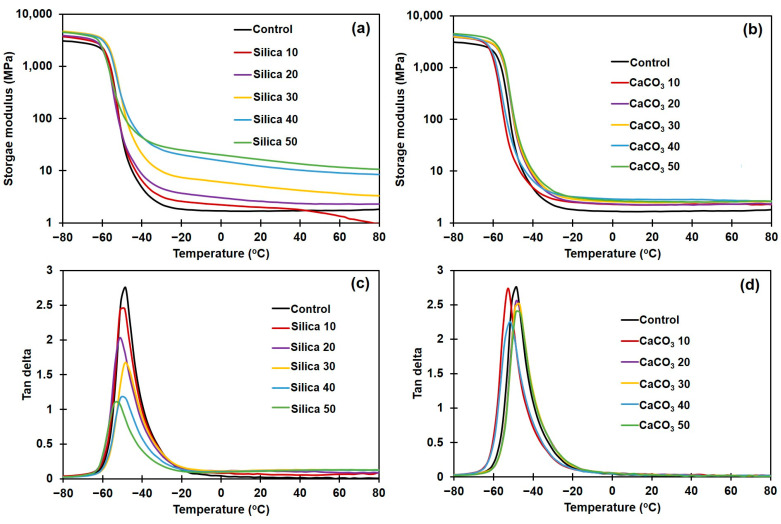
Dynamic mechanical properties of the rubber composites. (**a**) Storage modulus, and (**b**) tan delta of silica-filled composites; and (**c**) storage modulus, and (**d**) tan delta of CaCO_3_-filled composites.

Figure 8a,b display the E′ of silica- or CaCO_3_-filled NR composites, and Figure 8c,d show the tan delta for them. The raw data on E′ in the rubbery region, tan δ_max_, and T_g_ for the various composites are summarized in Table 5.

As shown in Figure 8a,b, the E′ in all cases decreased with temperature, reflecting the loss of material stiffness with temperature increase [48]. The E′ in rubbery plateau region continuously increased with filler loading for both filler types, due to the increased stiffness contributed by the fillers (Table 5). This result is in good agreement with the increases in M_H_ and M_H_ − M_L_ obtained in the curing test, previously illustrated in Figure 1 and Table 2. The NR composites became more rigid as a result of the higher filler loading [49,50]. These two methods (rheometric and DMA tests) provided similar stiffness trends as they were both performed at a low strain. Therefore, increasing the filler loading increases E′ of the composite. The effects of filler type and concentration on T_g_ of the rubber composites, as determined from the tan δ peaks, are shown in Figure 8c,d. The Tg and tan δ_max_ are also included in Table 5. It is seen that the T_g_ of NR shifted to a lower temperature upon incorporation of the fillers. Although this observation is somewhat unusual, this is not the first record of a decline in T_g_ for polymer composites. Previous studies have suggested that significant degradation of the polymer matrix during mixing with filler causes decreased T_g_ [51,52,53]. This may be the reason for T_g_ reduction in the current study, since the dumping temperature was quite high.

The tan δ_max_ of both types of NR composites decreased with the content of silica or CaCO_3_. Generally, the reduction in tan δ_max_ indicates the presence of strong rubber–filler interactions [54]. However, in some cases, a reduction in tan δ_max_ may not be related to the interactions between rubber and filler. Bandyopadhyaya et al. [55] reported that the height of tan δ peak decreased with filler content, indicating loss of the damping characteristics of the vulcanizates upon addition of the filler. The relative rigidity of fillers concerning the softer NR matrix is attributed to the loss of damping behavior in filled materials. Babal et al. [56] found that increasing the filler loading lowered the height of the tan delta peak because the contribution of the polymer matrix in T_g_ was reduced by the inclusion of the filler. This reasoning may be applicable in the current study, as the rubber–filler interactions and stress relaxation results were seemingly conflicting.

### 3.8. Strain-Induced Crystallization

**Figure 9:**
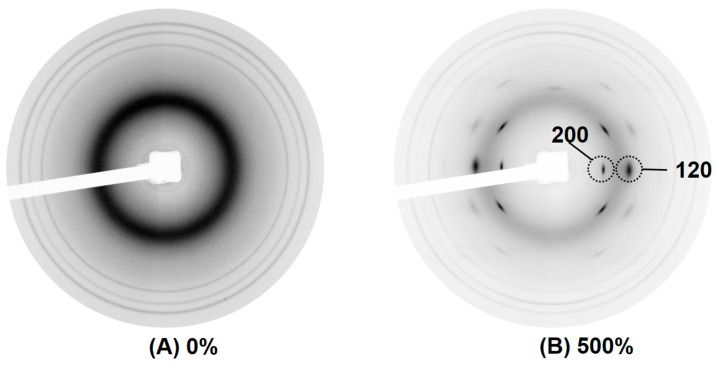
Typical 2D WAXS images of NR (control sample) in (**A**) unstretched state, and (**B**) in large tensile strain.

It is well recognized that the strain-induced crystallization (SIC) of NR is partly responsible for its outstanding properties. During stretching, the NR chains become oriented and aligned, forming crystallites [24]. These crystallites can be detected by X-ray diffraction techniques, here with Wide-angle X-ray scattering (WAXS). Typical two-dimensional (2D) WAXS profiles of NR at various strains are shown in Figure 9.

No reflection was observed in the sample without deformation (Figure 9A), indicating no crystallization had yet occurred. Upon deformation at 500% strain (Figure 9B), various reflection spots assigned to different crystallites were formed. Among the different reflection spots, the crystallographic planes (200) and (120) are of high interest.

**Figure 10:**
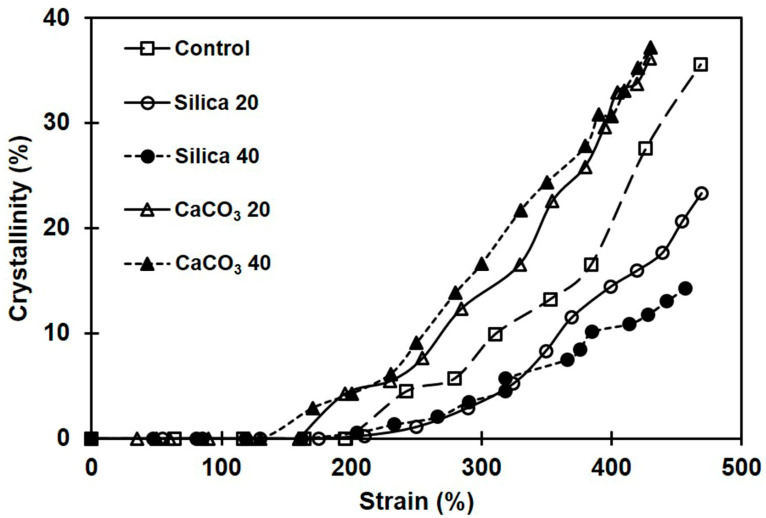
Crystallinity development during stretching of rubber composites filled with silica or with CaCO_3_.

Figure 10 shows the development of crystallinity in the various NR samples. The crystallinity of NR without filler is also included for comparison. The crystallinity in all cases increased with strain, confirming that NR crystallization was induced by stretching. Interestingly, the NR composite filled with CaCO_3_ exhibited higher crystallinity at any particular strain compared to both silica-filled composites and pure NR. The drastic enhancement of the crystallinity in the composites filled with CaCO_3_ was attributed to the comparatively strong rubber–filler interactions. Strong interactions between rubber and filler enhance the alignment of the NR chains in the stretching direction, resulting in increased crystallinity [18].

As for the silica-filled composites, insufficient rubber–filler interactions resulted from poor filler dispersion and hindered the orientation of rubber chains, suppressing crystallization. Therefore, compared to composites filled with CaCO_3_ and even pure NR, the total crystallinity of NR filled with silica filler was lower. The crystallinity reduction in the rubber composites with poor filler dispersion and weak rubber–filler interactions has been reported earlier [18,57].

It is also seen from Figure 10 that the strain where the first crystallinity was found was smaller for the composites filled with CaCO_3_ compared to others. This was tentatively attributed to the combined effects of the uniformity of CaCO_3_ dispersion and good rubber–filler interactions. The greater rubber–filler interactions between NR and CaCO_3_ enhanced overall crosslink density (Figure 4), shortening the distance between consecutive linkages along a polymer backbone. These shorter chains are easily oriented during stretching and serve as initiators of strain-induced crystallization at a comparatively small strain. The strongest tendency for SIC was observed in the composite containing 40 phr CaCO_3_, which case also had the greatest tensile strength. The SIC observations further corroborate the strong rubber–filler interactions between CaCO_3_ and NR. Aside from increasing crystallinity, good rubber–filler interactions can induce the SIC to start early [18].

**Figure 11:**
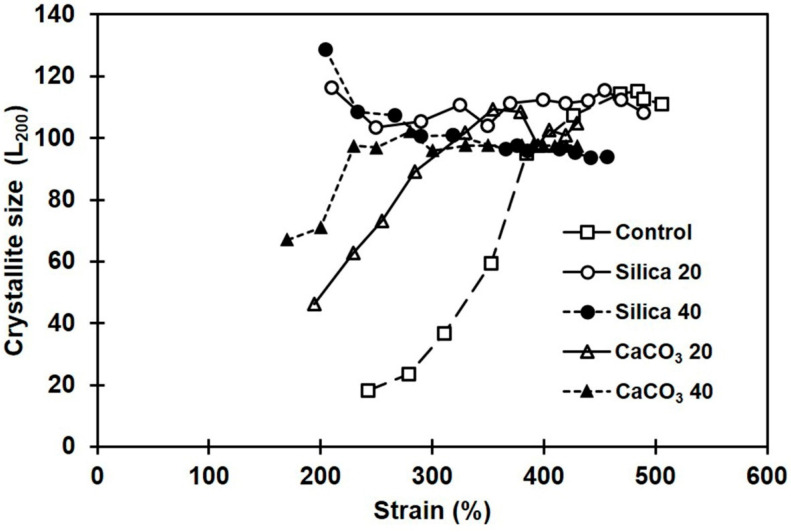
Crystallite sizes during stretching in the NR composites filled with silica or with CaCO_3_.

Influences of filler type and loading on crystallite size were also investigated. The crystallite sizes estimated from the 200-plane’s reflection are shown in Figure 11. Interestingly, the L_200_ showed opposite trends with the two fillers tested. The composites containing CaCO_3_ had increasing crystallinity with strain, whereas the silica showed a decrease in L_200_. The results clearly suggest that morphologies of the crystallites formed during deformation in the composites filled with CaCO_3_ and silica are somewhat different, which might be due to the different dispersion states, and differences in interactions between rubber and filler.

## 4. Conclusions

Effects of filler types, namely nano-sized silica and micrometer-sized CaCO_3_ and their respective content on mechanical and dynamic mechanical properties, and on strain-induced crystallization behavior of NR composites, were comparatively studied. Regardless of the filler type, increased filler loading increased curing torque and stiffness of the rubber composites. A more uniform filler dispersion was achieved with CaCO_3_ than with silica filler in NR, due to the lesser polarity of CaCO_3_. As result of the good filler dispersion, swelling resistance and rubber–filler interactions were enhanced by the increased overall crosslink density, and this also retarded the stress relaxation. The modulus and tensile strength increased with addition of either filler. The greatest tensile strength among the CaCO_3_-filled composites was achieved at 40 phr, with an improvement of about 88% over the unfilled sample. On the other hand, the highest tensile strength among the silica-filled NR was obtained at 20 phr, and it was only about 20% larger than that of the unfilled NR. This difference between the two fillers was attributed to the greater rubber–filler interactions that CaCO_3_ filler had. Strong rubber–filler interactions also enhanced strain-induced crystallization, lowering the onset strain for crystallization and increasing crystallinity during stretching. In contrast, large silica aggregates with poor dispersion were less restrictive to solvent penetration, and had comparatively ineffective rubber–filler interactions, reducing the overall crosslink density, and giving degraded thermomechanical properties, tensile properties, and ability of the NR matrix to crystallize during stretching. The results clearly suggest that CaCO_3_ should be favored over silica as the filler when producing some rubber products.

## Figures and Tables

**Table 1:** Formulation for preparation of composite compounds (phr is part(s) per hundred parts of rubber).

Chemical	Quantity (phr)	
	Silica	CaCO_3_
STR 20	100	100
ZnO	3	3
Strearic acid	1	1
TMQ	1	1
Filler	0, 10, 20, 30, 40 and 50	0, 10, 20, 30, 40 and 50
CBS	1.2	1.2
Sulfur	1.5	1.5

**Table 2:** Minimum torque (M_L_), maximum torque (M_H_), torque difference (M_H_ − M_L_), and cure rate index (CRI) for the rubber composites filled with silica or with CaCO_3_.

Sample\Property	M_L_ (dNm)	M_H_ (dNm)	M_H_ − M_L_ (dNm)	CRI (min^−1^)
Control	0.60	4.95	4.35	166.67
Silica 10	0.73	5.62	4.89	101.01
Silica 20	1.01	6.82	5.81	73.53
Silica 30	1.62	11.43	9.81	74.63
Silica 40	2.29	19.04	16.75	20.37
Silica 50	2.61	19.50	16.89	17.21
Control	0.60	4.95	4.35	166.67
CaCO_3_ 10	0.42	6.15	5.73	100.00
CaCO_3_ 20	0.48	7.29	6.81	78.50
CaCO_3_ 30	0.44	8.18	7.74	75.76
CaCO_3_ 40	0.54	9.28	8.74	73.00
CaCO_3_ 50	0.60	10.34	9.74	72.46

**Table 3:** Initial stress (σ_0_), and temperatures at which the force has dropped by 10%, 50%, and 90% (T_10_, T_50_ and T_90_) from the original force (F_0_), for rubber composites filled with silica or with CaCO_3_.

Parameter\Sample	Control	Silica 20	Silica 40	CaCO_3_ 20	CaCO_3_ 40
σ_0_ (MPa)	0.31	0.31	0.33	0.4	0.47
T_10_ (°C)	141.2	129.6	109.1	149.3	144.7
T_50_ (°C)	166.8	157.4	146	173.1	169.6
T_90_ (°C)	213.8	193.9	179.2	224.2	216.4
Slope value	−0.0125	−0.0253	−0.0371	−0.0086	−0.0092

**Table 4:** Tensile strength (TS), 100% modulus (100M), and elongation at break (EB) for rubber composites filled with silica or with CaCO_3_.

Sample\Property	100M (MPa)	TS (MPa)	EB (%)
Control	0.57 ± 0.02	9.24 ± 1.21	702 ± 39
Silica 10	0.43 ± 0.02	10.98 ± 0.15	697 ± 7
Silica 20	0.47± 0.03	11.11 ± 0.89	700 ± 13
Silica 30	0.49 ± 0.00	10.56 ± 0.48	686 ± 25
Silica 40	0.68 ± 0.08	9.99 ± 0.59	594 ± 33
Silica 50	0.87 ± 0.12	9.37 ± 0.27	586 ± 36
Control	0.57 ± 0.02	9.24 ± 1.21	702 ± 39
CaCO_3_ 10	0.74 ± 0.05	8.82 ± 0.98	466 ± 4
CaCO_3_ 20	0.84 ± 0.02	10.42 ± 0.60	477 ± 5
CaCO_3_ 30	0.71 ± 0.10	12.85 ± 0.79	681 ± 73
CaCO_3_ 40	0.80 ± 0.02	17.37 ± 0.72	663 ± 4
CaCO_3_ 50	0.86 ± 0.02	16.62 ± 0.18	648 ± 18

**Table 5:** Storage modulus (E′), tan delta peak height (tan δ_max_), and glass transition temperature (T_g_).

Sample	E′ (MPa)	tan δ_max_	T_g_ (°C)	Sample	E′ (MPa)	tan δ_max_	T_g_ (°C)
Control	1.68	2.76	−48	Control	1.68	2.76	−48
Silica 10	1.95	2.45	−49	CaCO_3_ 10	2.23	2.74	−53
Silica 20	2.50	2.02	−51	CaCO_3_ 20	2.25	2.54	−49
Silica 30	4.75	1.67	−49	CaCO_3_ 30	2.48	2.52	−47
Silica 40	11.66	1.17	−51	CaCO_3_ 40	2.84	2.25	−52
Silica 50	15.18	1.09	−54	CaCO_3_ 50	2.58	2.40	−49

## Data Availability

Data are contained within the article.
